# lncRNA APOC1P1-3 promoting anoikis-resistance of breast cancer cells

**DOI:** 10.1186/s12935-021-01916-w

**Published:** 2021-04-26

**Authors:** Qi Lu, Li Wang, Yabiao Gao, Ping Zhu, Luying Li, Xue Wang, Youping Jin, Xiuling Zhi, Jerry Yu, Xin Li, Xingjun Qin, Ping Zhou

**Affiliations:** 1 Department of Physiology and Pathophysiology, School of Basic Medical Sciences, Fudan University, No. 130 Dong’an Road, Shanghai, 200032 China; 2 Department of Oral & Maxillofacial – Head & Neck Oncology, Shanghai Ninth People’s Hospital, Shanghai Jiao Tong University School of Medicine, No. 639 Zhi Zao Ju Road, Shanghai, 200011 China; 3 Institutes of Biomedical Sciences, Fudan University, No. 130 Dong’an Road, Shanghai, 200032 China; 4 Center for Medical Research and Innovation, Shanghai Pudong Hospital, Fudan University Pudong Medical Center, 2800 Gongwei Road, Pudong, Shanghai, 201399 China; 5 Department of Medicine, University of Louisville, Louisville, KY 40292 USA

**Keywords:** Breast cancer, LncRNA APOC1P1-3, Anoikis resistance, MiRNA-188-3p, Tumor metastasis

## Abstract

**Background:**

Anoikis resistance plays a critical role in the tumor metastasis by allowing survival of cancer cells in the systemic circulation. We previously showed that long non-coding RNAs APOC1P1-3 (lncRNA APOC1P1-3) inhibit apoptosis of breast cancer cells. In this study, we explored its role in anoikis resistance.

**Methods:**

We induced anoikis resistance in two breast cancer cell lines (MCF-7 and MDA-MB-231) under anchorage-independent culture conditions and studied lncRNA APOC1P1-3 effects on apoptosis. Using Dual-Luciferase activity assay, we determined whether it specifically binds to miRNA-188-3P. We further explored its role in lung metastasis by injecting MDA-MB-231 and MDA-MB-231-APOC1P1-3-knock-down cells in female BALB/c nude mice.

**Results:**

We found that lncRNA APOC1P1-3 suppressed early apoptosis of these cells (demonstrated by gain or loss of their function, respectively) and promoted anoikis resistance via reducing activated- Caspase 3, 8, 9 and PARP. Moreover, it specifically binds to the target miRNA-188-3p acting as a “sponge” to block the inhibition of Bcl-2 (an anti-apoptosis protein).

**Conclusions:**

Our study supports a theory that lncRNA APOC1P1-3 can promote development of breast cancer metastasis via anoikis resistance by specifically binding to miRNA-188-3p to block the inhibition of Bcl-2.

**Supplementary Information:**

The online version contains supplementary material available at 10.1186/s12935-021-01916-w.

## Introduction

Breast cancer metastasis is a complicated multistep process involving cell detachment from the original site, survival in the circulatory and lymphatic systems, and colonization in distant target organs [1]. Anoikis is a particular type of apoptosis of epithelial and endothelial cells on losing attachment from the appropriate extracellular matrix or neighboring cells. It is a physiological process for organ development and tissue homeostasis [2]. Cancer cells usually acquire resistance to anoikis and become aggressive. Anoikis resistance is considered a necessary feature for metastasis. It plays a vital role in tumor survival via circulating tumor cells in the blood and facilitating secondary tumor formation in distal organs [3, 4]. A novel TDO2-AhR signaling axis promotes anoikis resistance when using global expression analysis and metabolomic profiling of triple-negative breast cancers (TNBC) cells in forced suspension [5]. Generally, anoikis resistance involves many intracellular molecules, such as growth factor receptors, microRNAs (miRNAs) and long noncoding RNAs (lncRNAs) [6].

LncRNAs are a set of key regulatory RNAs with transcripts longer than 200 nucleotides. They are involved in almost all physiological and pathological processes, including cell proliferation, apoptosis, stem cell maintenance, and cancer metastasis [7–10]. They directly regulate metastasis both in vitro and in vivo. LncRNA HOTAIR is aberrantly expressed in many tumors (liver, breast, colon and pancreatic cancers), contributing to metastasis [11, 12]. LncRNA MALAT1 was discovered first in non-small cell lung cancer patients with high metastasis and poor prognosis [13]. It is also upregulated in prostate, colorectal, and bladder cancers [14–16]. lncRNAs operate under various mechanisms, including sponge of miRNA, epithelial-mesenchymal transition and epigenetic regulations [17]. However, their mechanisms in the anoikis resistance of breast cancer remain unclear. In our previous studies, we sought out long intergenic non-coding RNA APOC1P1-3 from breast cancer tissues and cell lines. It was upregulated in most cases and correlated with tumor size and hypomethylation in its promoter region. Moreover, it promoted cell proliferation and inhibited apoptosis via α-tubulin acetylation [18]. In the present study, we further explored its role in the anoikis resistance of breast cancer.

## Materials and methods

### Human breast cancer tissues and cell lines

A total of 24 freshly resected breast cancer samples and paired paracancerous tissues were collected from the Fudan University Shanghai Cancer Center. Our present research has been approved by the Basic Medical Ethics Committee of the Fudan University (2017-F001). The patient signed an informed consent. Specimens were snap-frozen in liquid nitrogen immediately after resection and kept in − 80 °C.

Twenty-four invasive ductal breast cancer patients who accepted modified radical mastectomy (Auchincloss), were included in this study. All patients were not subjected to chemotherapy and radiotherapy before surgery, except one with suspected lung metastasis: none had distant metastasis. 16 out of 24 (66.7%) cases had tumors with a maximum diameter above 2.5 cm. Histological grades were from II to III, and clinical stages were from I to III, according to the WHO classification of breast tumor released in 2012. Two pathologists were invited to review the hematoxylin and eosin (H&E) slides. The details of breast cancer patients are found in the Additional file 2: Table S5.

Breast cancer cell lines (MCF-7, MDA-MB-231, MCF-10A and T47D) were obtained from the cell bank of the Shanghai Institute of Biological Sciences, Chinese Academy of Sciences. The cells were cultured in DMEM/F12 medium with 10% (v/v) fetal bovine serum (Gibco, USA). The medium contained 100 unit penicillin and 100 µg/ml streptomycin, at 37 °C with 5% CO_2_.

### Cytoplasmic and nuclear separation

The content of RNA in the cytoplasm and nucleus was analyzed with PARISTM Kit (Thermo Fisher AM1921). When the confluence rate was >90% in the 60 mm dish, the cells were collected. Cytoplasm and nuclear materials were separated by centrifugation following the manual of kit. The cytoplasm supernatant and nucleus precipitate were transferred to different tubes with equal volumes of 2× Lysis Binding Buffer and mixed thoroughly with a pipette. The obtained RNA was stored at -80°C with an appropriate amount of DNAase to prevent degradation.

### RNA-Chip and bioinformatics analysis

The expression of lncRNA was screened by pairwise random variance in the cancer and control tissue samples. The specific criterion was 1.5 times higher lncRNA expression in cancer tissue, P ≤ 0.05. Relevant hierarchical clustering analysis was assisted by Kang Cheng Biotechnology Co, Ltd., using Cluster Treeview software (Standford).

### Real-time quantitative PCR

Total RNAs of samples were extracted by TRIzol from Invitrogen. Reverse transcription was performed with TOYOBO's ReverTra Ace qPCR RT Kit. Real-time PCR was performed with Bio-RAD iTaqTM Universal SYBR® Green Supermix. The primer sequences are described in Additional file 1: Table S4.

### Anoikis model of breast cancer cells

Anoikis model was established by poly-2-hydroxycthyl methacrylate (polyHEMA) (Sigma company) culture. PolyHEMA was dissolved in ethanol (final concentration of 120 mg/ml) in a 65 °C water bath. The PolyHEMA solution (1:10 in 95% ethanol) was sterilized under a 0.22 µm filter and pipetted into a 6-well culture plate (1 ml/well). Prior to use, the plate was incubated overnight at room temperature, washed three times with PBS, and incubated with DMEM/F12 medium (Thermo Fisher). 1 × 10^6^ cells were seeded into the PolyHEMA-coated wells for 12, 24, 48 or 72 h in a humidified (37 °C, 5% CO_2_) incubator.

### Annexin V-FITC/PI double staining for apoptosis

MDA-MB-231a and MCF-7a cells cultured for 48 h were collected, digested with 0.25% EDTA-free trypsin, and pipetted to achieve a uniform single cell suspension. Cell concentration was adjusted to L-15/DMEM medium containing 10% FBS, 10% double antibiotics to 1 × 10^6^cells/ml. Annexin V-FITC/PI double staining was performed with the Annexin V、FITC Apoptosis Detection Kit (Dojindo).

### Dual-Luciferase report gene vectors

The pmirGLO Dual-Luciferase miRNA Target Expression Vector and Dual-Luciferase® Reporter Assay were purchased from Promega. Using bioinformatics to analyze and predict the binding site of miRNA-188-3p to APOC1P1-3, we designed primers and cloned the target fragment. The fragment was inserted into the pmirGLO Dual-Luciferase miRNA Target Expression Vector by molecular cloning, and the recombinant reporter plasmid and the target microRNA mimic were co-transfected into the target cells. The luciferase was extracted and its activity was tested. The mutated fragment was ligated into the dual luciferase reporter gene vector, and the target microRNA mimic was co-transfected with the target cell for luminescence detection. The DNA purification kit、Glue recovery kit and Rapid site-directed mutagenesis kit were purchased from TIANGEN. Endonuclease XhoI、SalI and T4 ligase were from NBE.

### Plasmid, siRNAs, microRNA mimic/inhibitor and transfections.

The pcDNA3.1-sense is the plasmid of cDNA encoded full-length lncRNA APOC1P1-3, which was PCR-amplified using primers (Additional file 1: Table S3). Amplification was performed for 35 cycles at 95 °C for 45 s, at 60 °C for 45 s, and at 72 °C for 1 min, and subcloned into Bam HI and Xho I sites of a pcDNA3.1 vector (Invitrogen, Carlsbad, CA, USA). Transfections for pcDNA3.1/APOC1P1-3, siRNA-196 (Ribobio), miRNA-188-3p mimic/inhibitor (Ribobio) were performed using the Lipofectamine™ 3000 Transfection Reagent (Invitrogen) with Opti-MEM (Gibco, USA) according to the manufacturer's instructions.

### Western blot

The cells were collected and lysed in RIPA lysis buffer (Thermo Fisher, cat. 89,900, USA). The protease inhibitor cocktail (Roche, 11836153001) was added into the lysis buffer. Protein concentrations were determined by a Pierce™ BCA Protein Assay Kit (Thermo Fisher, 23225, USA). Equal amounts of proteins (20 μg) were loaded on sodium dodecyl sulfate polyacrylamide gel electrophoresis (SDS-PAGE) gel and transferred onto 0.45 μm polyvinylidene difluoride (PVDF) membranes (Merck Millipore, USA). Then, the membranes were blocked with 5% non-fat milk and probed with primary and secondary antibodies (The Caspase-3,8,9 and active Caspase-3,8,9 antibodies ordered from Cell signaling technology, PARP and cleaved-PARP ordered from Abcam; the secondary antibodies from Thermo Fisher).

### In vivo metastasis studies

We firstly designed a lentivirus for knock-down human APOC1P1-3 (5′ GAAAGACCCTGGAGGACTA 3′) from GeneChem Corporation (Shanghai, China). We seeded 2 × 10^5^ MBA-MD-231 cells in 6-well plates and carried out the transfection with Lv-sh-APOC1P1-3 and Lv-sh-Control 24 h. At 72 h, we extracted total RNA from the cells and evaluated the inhibitory effects (about 60%) with RT-PCR (Additional file 1). Then, we established the APOC1P1-3 knock-down and control stable MDA-MB-231 cell lines with transfected Lv-sh-APOC1P1-3, Lv-sh-Control and the Puromycin (2.5 μg/ml).

To test the role of APOC1P1-3 in lung metastasis of breast cancer cells in vivo, we injected the MDA-MB-231 and MDA-MB-231 with APOC1P1-3 knock-down cells (2 × 10^6^) into the tail vein of 6-week-old female BALB/c nude mice (8 mice/group). Seven weeks following the inoculation, the mice were sacrificed and lung samples were harvested for quantifying the metastatic sites. Hematoxylin and Eosin (HE) staining was used to analyze the distribution of cancer cells in the lungs. All animal experiments were performed in accordance with the guidelines and approved by the Institutional Animal Care and Use Committee at Fudan University (20,160,225–108).

### Statistical analyses

All values are reported as mean ± SE and analyzed with the SPSS 17.0 (Chicago, IL, USA). For comparisons, one-way analyses of variance, Fisher’s exact tests, X^2^-tests, and two-tailed students’ t-tests were performed. P ≤ 0.05 was considered to be statistically significant. The diagrams were completed with Prism 5.0 (GraphPad Software, La Jolla, CA, USA).

## Results

### Characterization of lncRNA APOC1P1-3

Previously, we measured and compared lncRNA APOC1P1-3 expression in breast cancer and its adjacent tissues via microarray chip [18]. In order to identify its protein-noncoding property, we checked the Open Reading Frame (ORF) of APOC1P1-3 by ORF finder (http://www.ncbi.nlm.nih.gov/orffinder/), and found that it has 8 ORFs: none is more than 200nt, suggesting no protein coding potential (Additional file 1: Table S1). CSF is the mutation rate of the codon. Codons are relatively conservative in the coding region, but prone to mutations in the non-coding region. Due to the problem of sequence conservation, Michael F Lin [19] proposed a new value to introduce the evolution model PhyloCSF. We also checked the PhyloCSF of APOC1P1-3 on UCSC (http://genome.ucsc.edu/). All values in the exon of APOC1P1-3 are less than 0, indicating no conservative type.

### Expression of lncRNA-APOC1P1-3 in breast cancer

**Fig. 1:**
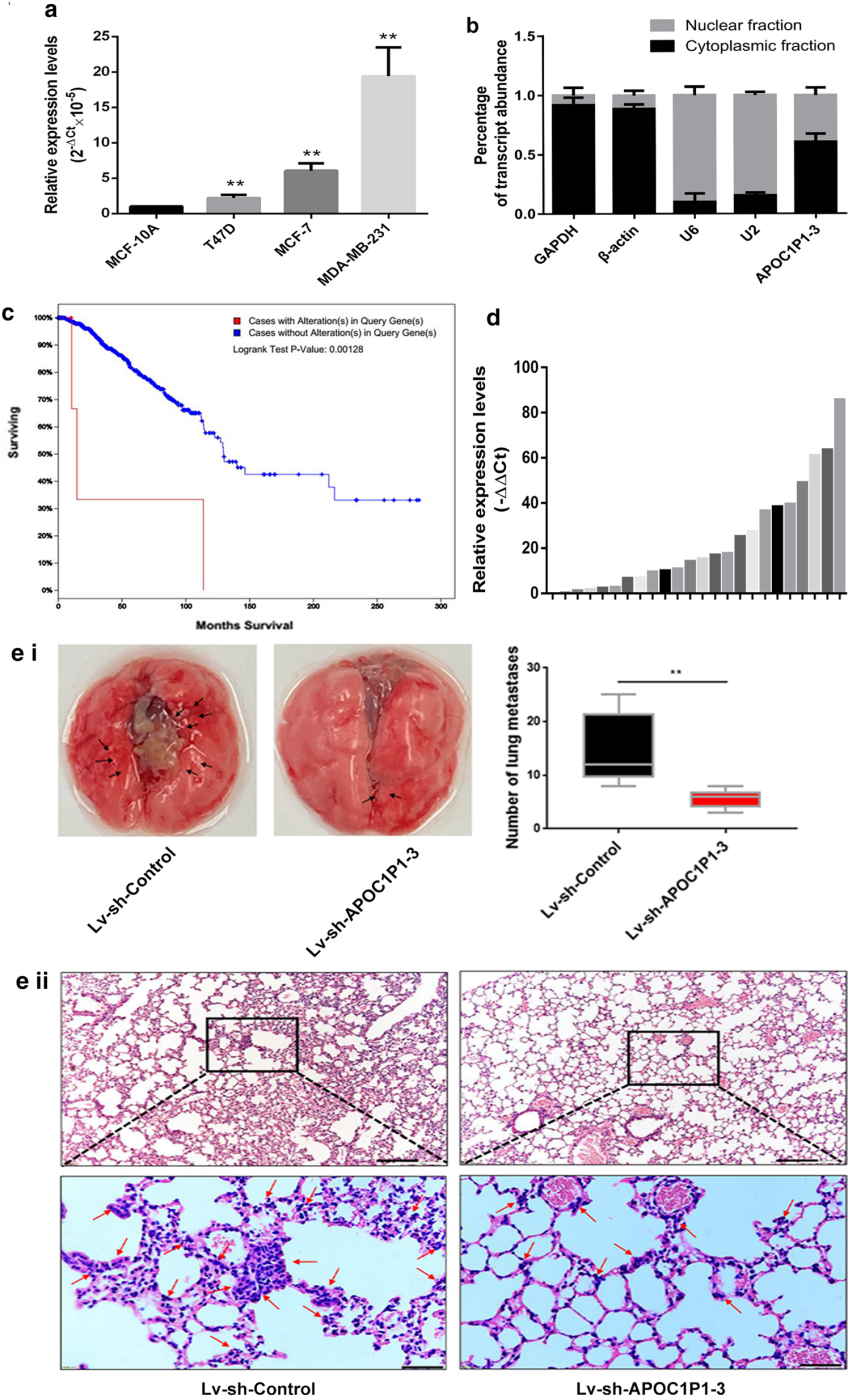
Correlation between lncRNA-APOC1P1-3 and breast cancer. **a** The lncRNA-APOC1P1-3 expression in breast cancer cells (T47D, MCF-7, MDA-MB-231) and normal mammary epithelial cells (MCF-10A) (real time-PCR). n = 3, relative to the control group**P < 0.01. **b** LncRNA-APOC1P1-3 expressed in both cytoplasm and nuclear compartments in MDA-MB-231 cells (real time-PCR). U6 and U2 RNA were used for nuclear gene expression; and GAPDH and β-actin RNA were for cytoplasmic gene expression. **c** The overall survival rate of breast cancer patients. Red line, high expression of APOC1P1-3 (110 months), blue line, low expression of APOC1P1-3 (300 months) (n = 816, P < 0.01). **d** The expression of lncRNA-APOC1P1-3 in 24 fresh breast cancer tissues (RT-PCR). **e** APOC1P1-3 promotes lung metastasis. (i) The number of lung metastases after tail vein injection with MDA-MB-231 cells infected with APOC1P1-3-knock-down or control vector. (ii) Representative Hematoxylin and Eosin stain (H&E) of lung metastases. Scale bars: above picture is 200 μm, below picture is 50 μm. **, P < 0.01

We analyzed the expression of lncRNA APOC1P1-3 in one normal (MCF-10A), three malignant (T47D, MCF-7, MDA-MB-231) breast cell lines, and 24 breast cancer tissues by real-time qPCR (Fig. 1a, d). LncRNA APOC1P1-3 expression was upregulated in malignant cell lines, especially in the MDA-MB-231 cells (increased by 1.5-fold). In addition, lncRNA APOC1P1-3 was found in both the cytoplasm and nucleus in MDA-MB-231 cells (Fig. 1b).

To evaluate the role of lncRNA APOC1P1-3 in patient survival, we used the cBioPortal database in TCGA for related bioinformatics analysis. The survival rate is much higher in patients with low expression (blue line) than with high expression lncRNA APOC1P1-3 (red line) (Fig. 1c; n = 816, P < 0.01). This may be related to distant metastasis. We further explored the role of APOC1P1-3 in the lung metastasis by injecting MDA-MB-231 and MDA-MB-231-APOC1P1-3-knock-down cells into the mouse tail vein. The results showed that the lung metastases burden in nude mice of APOC1P1-3-knock-down cells significantly decreased (Fig. 1e (i), e(ii)). The metastatic sites in wild MDA-MB-231 are more than MDA-MB-231-APOC1P1-3-knock-down cells. This indicates that the lncRNA APOC1P1-3 may promote the distant metastasis.

### The capacity of anoikis resistance of breast cancer cells

**Fig. 2:**
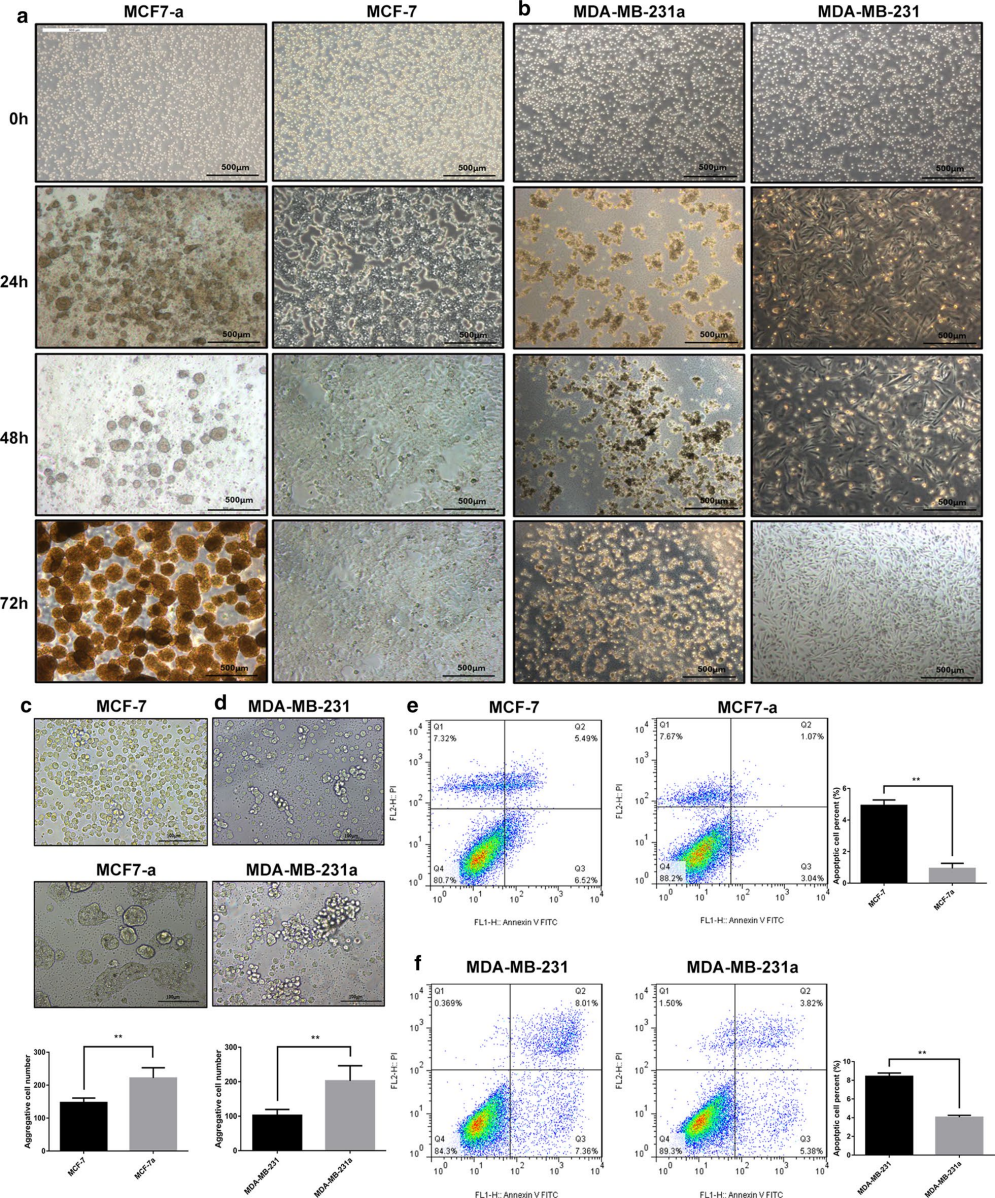
Morphological changes, aggregation rate and apoptotic changes of anoikis resistant cells. **a**, **b** MCF-7a/MDA-MB-231a cells were cultured in anoikis model for 24, 48 and 72 h. MCF-7/MDA-MB-231 cells were cultured in adherent state as normal control. As the induction time of MCF-7a/MDA-MB-231a increases in the anoikis model, suspended single breast cancer cells begin to aggregate gradually. **c**, **d** Microscopic morphology of MCF-7a/MDA-MB-231a and MCF-7/MDA-MB-231 cells 12 h culture in anoikis or normal groups. The aggregation rate in MCF-7a/MDA-MB-231a is significantly more than MCF-7/MDA-MB-231 groups (n = 7, **P < 0.01). **e**, **f** The effect of anoikis resistant on MCF-7a/ MDA-MB-231a cells (Annexin V-FITC/PI double staining). Early apoptosis was significantly reduced in the MCF-7a/MDA-MB-231a group than in the MCF-7/MDA-MB-231 group (n = 3, **P < 0.01)

The anoikis resistance model was established by culturing the cells on a low-adherent culture plate with polyHEMA [20]. Both MDA-MB-231 and MCF-7 cells survived for 72 h under adherent and anchorage-independent conditions by forming micro-tissues (Fig. 2a, b), indicating anoikis resistance. MCF-7a aggregated in large clusters, with more cells were dead than MDA-MB-231a, which is similar to a previous report [21]. MDA-MB-231a cells formed many small irregular clumps, which may be responsible for detachment from primary tumor tissues.

Under both adherent culture and ultralow attachment culture, we collected cells with or without anoikis resistance for 48 h. Aggregation occurred in all cells (MCF-7a, Fig. 2c and MDA-MB-231a, Fig. 2d). The aggregation was much more in the anoikis group than in the normal group (n = 7, **P < 0.01).

We compared the number of apoptotic cells under adherent culture and low-adherent culture condition by Fluorescence-activated cell sorting (FACS). After 48 h culture, the numbers of apoptotic cells were significantly fewer in MCF-7a and MDA-MB-231a (Fig. 2e–f) than in MCF-7 and MDA-MB-231 groups (n = 3, **P < 0.01), indicating an effective induction of anoikis resistance.

### LncRNA APOC1P1-3 suppressing apoptosis via apoptosis-related proteins

**Fig. 3:**
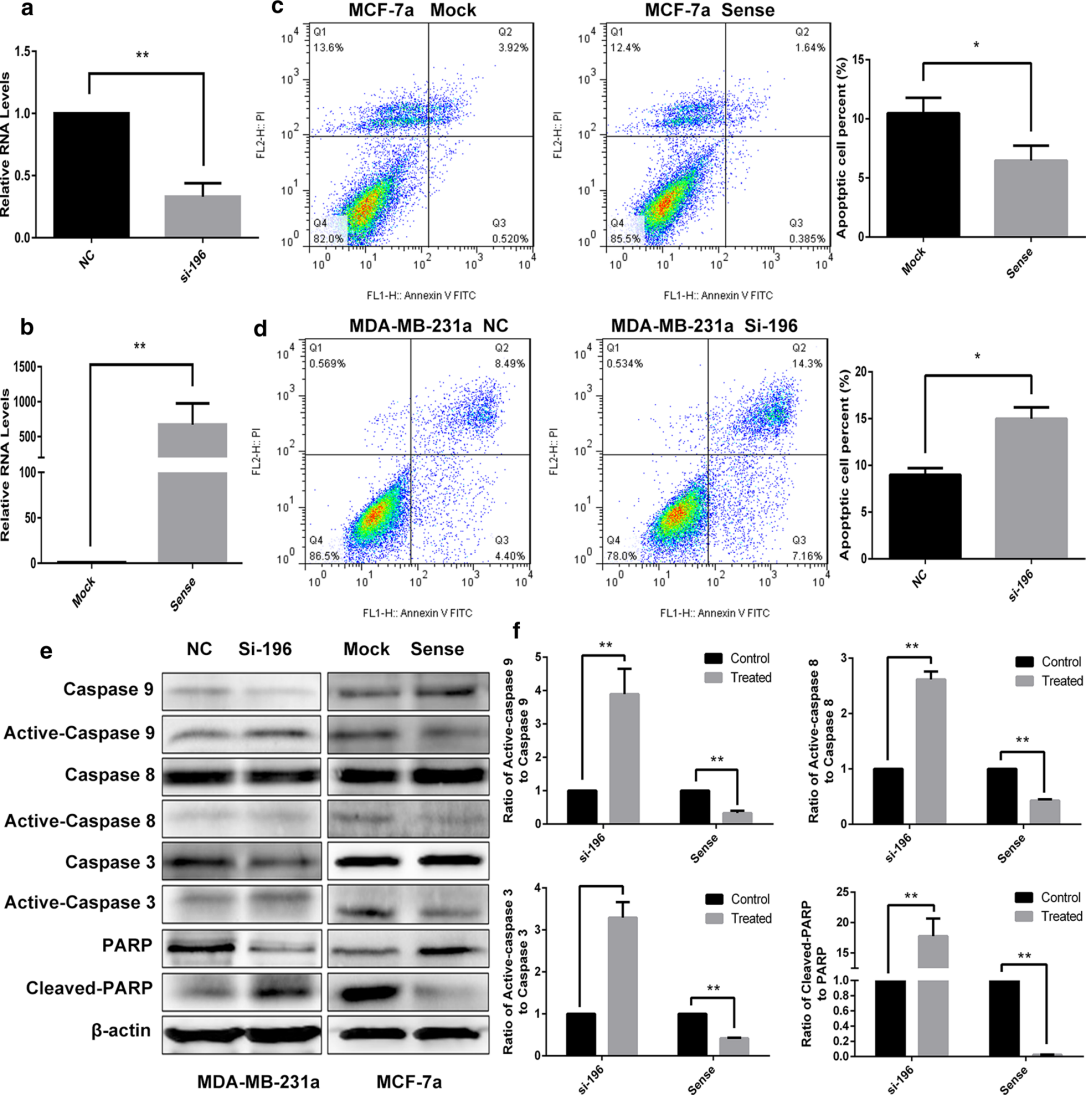
Effects of lncRNA-APOC1P1-3 on anoikis resistant. **a**, **b** The expression of APOC1P1-3 was inhibited in MDA-MB-231 cells transfected with siRNA-196, but increased in MCF-7 cells transfected with pcDNA3.1-sense (qRT-PCR) (n = 3, **P < 0.01). **c**, **d** Early apoptosis was more in MDA-MB-231a cells (low expression of APOC1P1-3) than in MCF-7a cells (high expression). **e**, **f** Apoptosis-related protein (Western Blot). Similarly, in MDA-MB-231a cells active–Caspase 3, 8,9 and cleaved-PARP expressions and the shear rate of apoptotic proteins increased, but decreased in MCF-7a cells

In the gain/loss functionality studies, the expression of lncRNA APOC1P1-3 efficiently up-regulated by transfection with pcDNA3.1-sense in MCF-7 cells and down-regulated by transfection with siRNA-196 in MDA-MB-231 cells (Fig. 3a, b). The percentage of early apoptosis (FACS) was significantly decreased in MCF-7a cells (Fig. 3c) (n = 3, *P < 0.05), and increased in MDA-MB-231a cells (Fig. 3d) (n = 3, *P < 0.05). The results support that the lncRNA APOC1P1-3 could promote anoikis resistance of breast cancer cells.

To probe the mechanisms of anoikis resistance, we measured apoptosis related proteins (Western Blot) from MDA-MB-231a transfected with siRNA-196, as well as MCF-7a transfected with pcDNA3.1-sense (Fig. 3e–f) (n = 3, **P < 0.01). The expressions of activated-caspase 3, 8, 9, and cleaved-PARP upregulated in MDA-MB-231a cells, which were knockdown with siRNA-196. In contrast, the expressions decreased in MCF-7a cells, which were overexpressed with pcDNA3.1-sense. These results indicate that upregulation of lncRNA APOC1P1-3 could influence anoikis resistance via caspase-dependent pathway.

### Regulation of anoikis resistance via binding to miRNA-188-3p

**Fig. 4:**
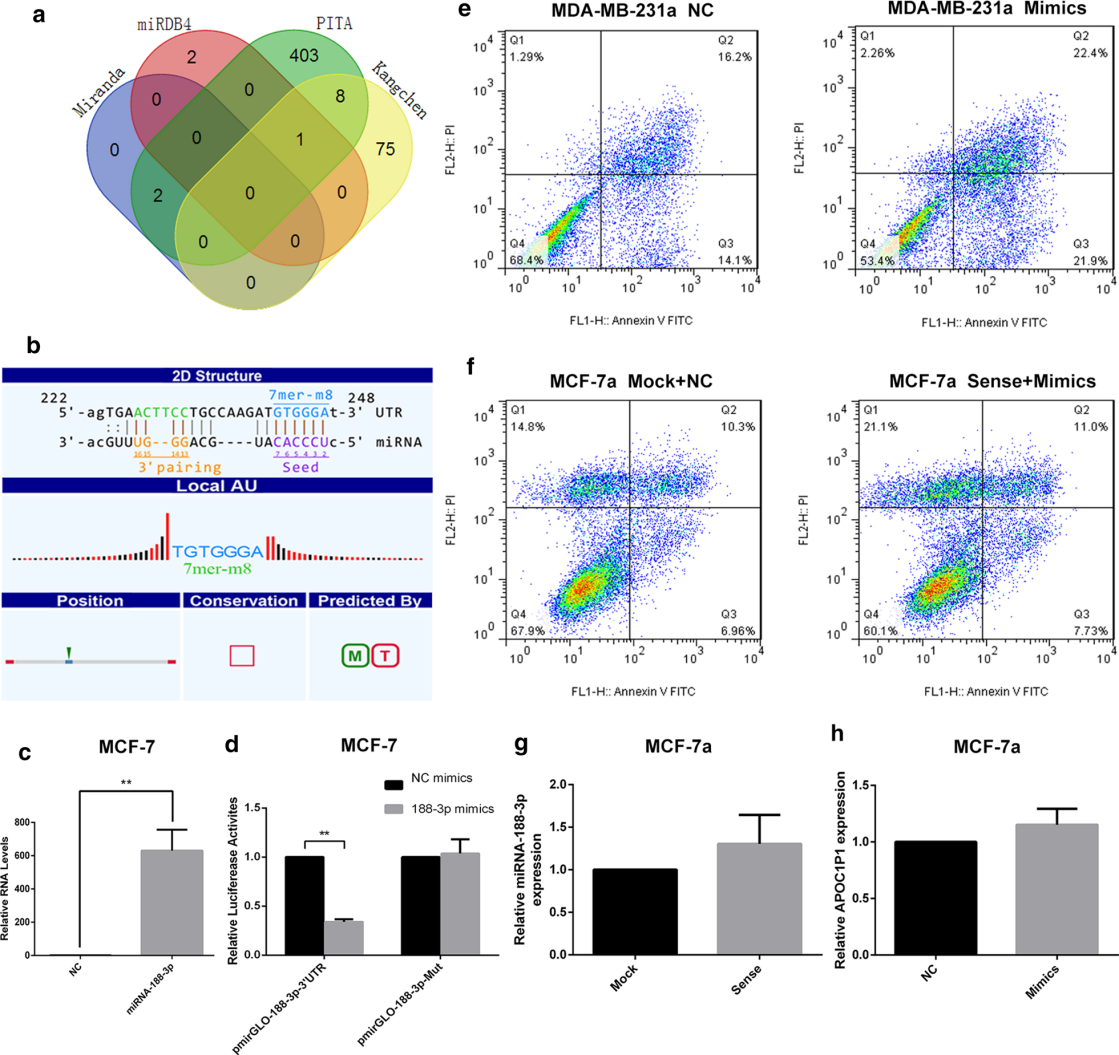
lncRNA-APOC1P1-3 binds to miRNA-188-3p, and miRNA-188-3p reverses the anti-apoptotic effect of LincRNA-APOC1P1-3. **a** Venn diagram of target microRNAs predicted by multiple databases. **b** RNA-chip predicted binding sites of APOC1P1-3 with miRNA-188-3p. **c** The overexpression of transfected miRNA 188-3p mimics was detected by qPCR, which was significantly higher than that in the control group (n = 3, **P < 0.01). **d** Combination of APOC1P1-3 with miRNA-188-3P. When the pmirGLO-APOC1P1-3′UTR plasmid and miRNA-188-3P mimics were co-transfected, the luminescence signal significantly decreased. When the pmirGLO-APOC1P1-Mut plasmid and miRNA-188-3P mimics were co-transfected, the luminescence signal was restored. **e** Compared with the control group, early apoptosis of MDA-MB-231a cells transfected with miRNA-188-3p mimics increased significantly (FACS). **f** Overexpression of miRNA-188-3p followed by overexpression of lncRNA- APOC1P1-3 reversed its pro-apoptotic effect(FACS). **g**, **h** There’s no difference in the expression level of lncRNA- APOC1P1-3 after overexpressing miRNA-188-3p (qRT-PCR). After overexpressing lncRNA- APOC1P1-3, the expression of miRNA-188-3p was also unchanged

We used RNA microarrays (provided by Kangcheng Company) to screen related target microRNAs of lncRNA-APOC1P1-3 in breast cancer tissues, and predicted microRNAs in multiple databases like PITA, MIRDB4.0 and Miranda, by matching between sequences (Additional file 1: Table S2). There are 84 microRNAs that may bind to APOC1P1-3. With Venn diagram to compare the predicted results of databases and chip, miRNA-188-3p most likely binds to lncRNA APOC1P1-3 (Fig. 4a). The microarray results suggest that the binding site is at 222 bp-248 bp of APOC1P1-3 (Fig. 4b).

The pmirGLO-APOC1P1-3′UTR and the pmirGLO-APOC1P1-Mut vectors were separately constructed with the Dual-Luciferase Report Gene System. We used the BiBiServ-RNAhybrid database to perform thermodynamic analysis of the predicted binding sites before making a point mutation. The mfe value of the predicted site is -28.5 kcal/mol (Additional file 1: Figure S1), the highest absolute value among all 11 predicted sites, indicating a good thermodynamic effect. Group 4 mfe random mutation was -25.4 kcal/mol (Additional file 1: Table S3), indicating a better thermodynamic effect.

By transfected miRNA 188-3p mimics, we found that the expression of miRNA-188-3P was significantly higher than that in the control group (Fig. 4c). The pmirGLO-APOC1P1-3′UTR plasmid was bound to the co-transfected miRNA-188-3P mimics. Since the transcription of the luciferase reporter gene was blocked, the luminescence was significantly lowered. After mutation (Additional file 1: Figure S2), miRNA-188-3P mimics could no longer bind to it, and the luciferase reporter gene was transcribed normally (Fig. 4d). The luminescence was not significantly different from the control, suggesting that miRNA-188-3P can specifically bind to lncRNA-APOC1P1-3.

### The cross-talking between miRNA-188-3p and lncRNA-APOC1P1-3

We further explored the effect of miRNA-188-3p on anoikis resistance. miRNA-188-3p was first overexpressed in MDA-MB-231a cells by transfection with miRNA-188-3p mimics. Early apoptosis was highly increased after the overexpression (Fig. 4e), suggesting an inhibition of anoikis resistance. After co-overexpression of lncRNA APOC1P1-3 and miRNA-188-3p, there was no significant difference in early apoptosis between Sense + Mimics and the controls (Fig. 4f), revealing that miRNA-188-3p can reverse the effect of lncRNA-APOC1P1-3 in anoikis resistance.

Moreover, we studied the mutual regulation between lncRNA APOC1P1-3 and miRNA-188-3p. Firstly, we overexpressed APOC1P1-3 in MCF-7a cells by transfection with pcDNA3.1-sense, and evaluated the expression of miRNA-188-3p, and vice versa. Neither overexpression of miRNA-188-3p nor lncRNA-APOC1P1-3 caused a change (Fig. 4g, h). Thus, although lncRNA APOC1P1-3 binds to miRNA-188-3p, the two molecules do not have any mutual impact.

### LncRNA-APOC1P1-3 synergizes with miRNA-188-3p to affect Bcl-2

**Fig. 5:**
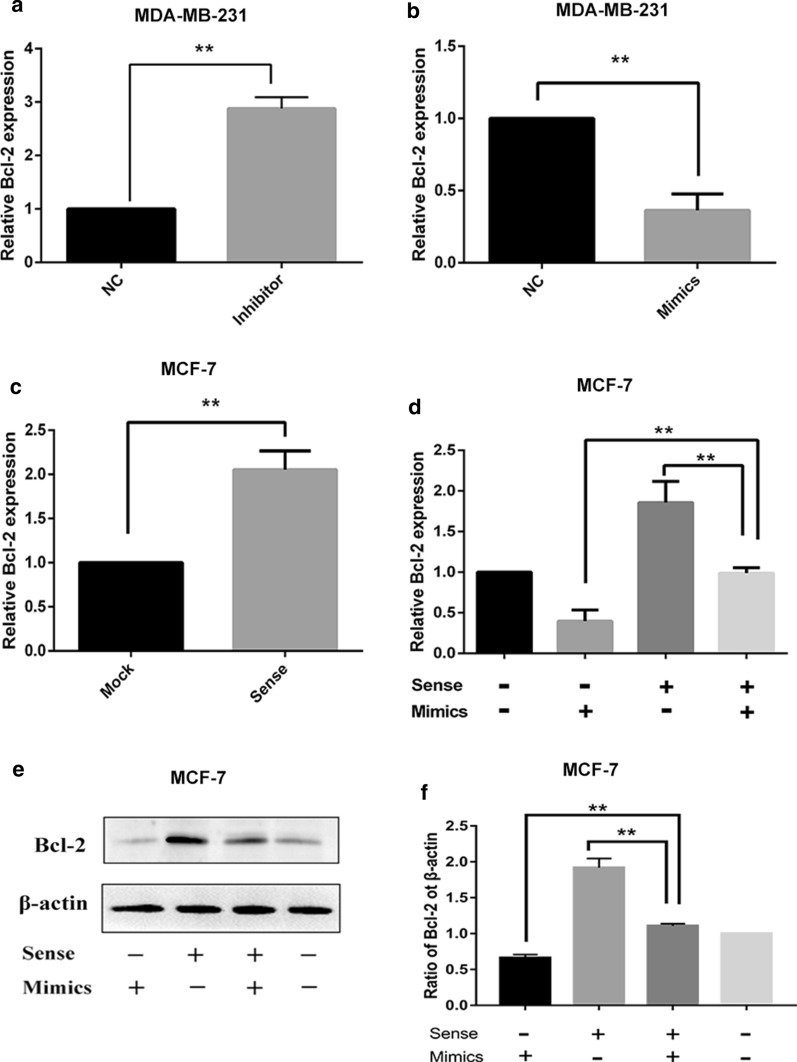
Effects of LncRNA- APOC1P1-3 and miRNA-188-3p on the expression of Bcl-2. **a**, **b** The down-regulation of miRNA-188-3p in breast cancer cells promotes Bcl-2 gene expression, but overexpression of miRNA-188-3p decreases the Bcl-2 gene expression; **c** Overexpression of lncRNA- APOC1P1-3 increases the expression of Bcl-2. **d** Overexpression of lncRNA- APOC1P1-3 followed by up-regulation of miRNA-188-3p abolished the lncRNA- APOC1P1-3 effect on Bcl-2. **e**, **f** In four groups (sense + /mimics-, sense-/mimics + , sense + /mimics + , sense-/mimics-) simultaneous transfection of sense and mimics reversed the expression of Bcl-2 when separately transfected with sense/mimics

We firstly found that the expression of Bcl-2 increased when transfected the inhibitor of miRNA-188-3p (n = 3, **P < 0.01) (Fig. 5a). Conversely, overexpression of miRNA-188-3p decreased Bcl-2′s level (n = 3, **P < 0.01) (Fig. 5b). Overexpression of lncRNA-APC1P1-3 increases the expression of Bcl-2 (n = 3, **P < 0.01) (Fig. 5c). Subsequently, to study the interaction of lncRNA APOC1P1-3 and miRNA-188-3p on the anti-apoptotic protein Bcl-2, we examined the Bcl-2 expression in 4 groups (sense + /mimics-, sense-/mimics + , sense + /mimics + , sense-/mimics-) (Fig. 5d). Overexpression of lncRNA APC1P1-3 (sense + /mimics-), significantly increased Bcl-2 (n = 3, **P < 0.01), while overexpression of miRNA-188-3p (sense-/mimics +) decreased it (n = 3, **P < 0.01). Interestingly, overexpression of both lncRNA APOC1P1-3 and miRNA-188-3p (sense + /mimics +) did not alter Bcl-2 expression (n = 3, **P > 0.01). We obtained the same result with Western Blot (Fig. 5e, f). Thus, APOC1P1-3 may enhance the anoikis resistance by blocking the inhibition of miRNA-188-3p against the Bcl-2.

## Discussion

**Fig. 6:**
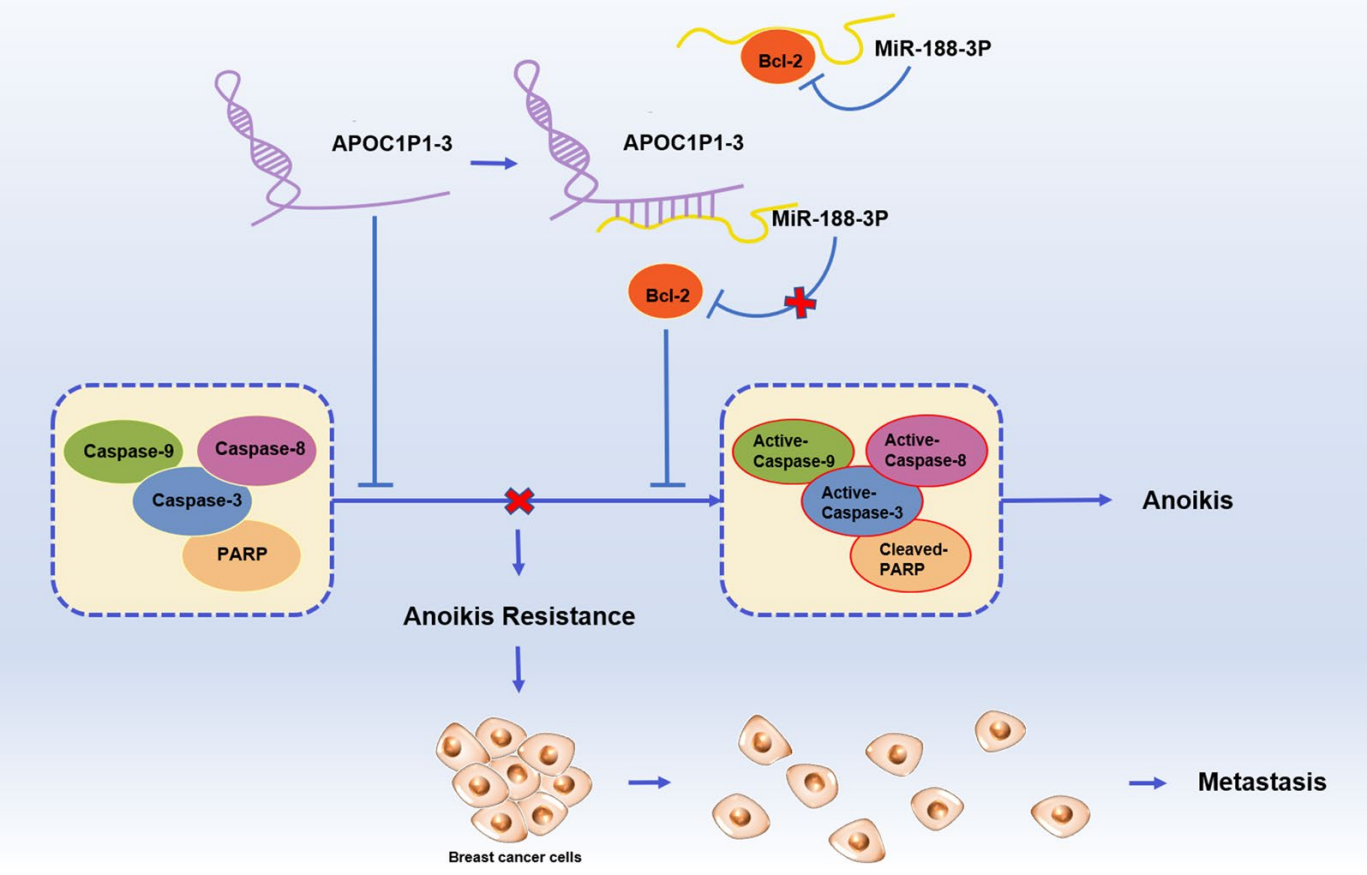
The molecular mechanism of LncRNA- APOC1P1-3 in anoikis resistance. Two pathways, lncRNA APOC1P1-3 inhibits Caspase 3, 8, 9 and PARP, and specifically binds to miRNA-188-3p to block its inhibition of Bcl-2.

Increasing evidence indicates the importance of lncRNAs in breast cancer metastasis [22–24]; however, the relation among specific lncRNAs and anoikis resistance and distant metastasis remains undetermined. Previously, we found lncRNA APOC1P1-3 is related to breast cancer. Thus, we focused on its role in anoikis resistance and regulatory mechanisms in the present study. We established an anoikis resistance model in both MDA-MB-231 and MCF-7 cell lines by low-adherent culture. The numbers of apoptotic cells were fewer in anoikis resistance groups than control groups. lncRNA APOC1P1-3 suppressed early apoptosis (MCF-7 and MDA-MB-231) in the anoikis resistance model by inhibiting active Caspase 3, 8, 9 and cleaved PARP. The animal experiment also demonstrated that the lncRNA APOC1P1-3 promotes lung metastasis of breast cancer cells (MDA-MB-231). Furthermore, we explore the mechanism of the lncRNA APOC1P1-3 in the anoikis resistance. miRNA-188-3p is the potential target molecule of lncRNA APOC1P1-3 (bioinformatics analysis and Dual-Luciferase report system) (Fig. 6).

Anoikis resistance is one of the malignant phenotypes in breast cancer metastasis [21], involving many factors. Higher PRKCQ/PKCθ expression can promote growth-factor-independent growth, anoikis resistance, and migration in triple-negative breast cancer cells [25]. Kaiso target gene Wnt11 induces anoikis resistance of metastasis in mouse lobular breast cancer cells [26].

Accumulating evidence suggests that lncRNAs could regulate the anoikis resistance to promote metastasis [27]. Indeed, Seitz et al. reported that lncRNA LINC00958 significantly reduced anoikis resistance in FL3 cell after knock-down [28].

Evidence suggests that lncRNAs are involved in breast cancer evolution, which can be divided into oncogene and tumor suppressors. Many lncRNAs have been suggested as molecular markers for predicting progression into invasive form. For instance, the lncRNA genes (LINC00324, PTPRGAS1 and SNHG17) have been related to ER^+^ and ER^−^ subtypes, tumor histologic grade and clinic outcomes [22]. In a previous study, we found lncRNA APOC1P1-3 to be aberrantly overexpressed in breast cancer, and correlated with the tumor size and hypomethylation in its promoter. It decreased α-tubulin acetylation and apoptosis. In this study, we hypothesized that lncRNA APOC1P1-3 plays important role in the anoikis resistance in malignant breast cancer cells.

We established a model for testing the anoikis resistance of MDA-MB-231 and MCF-7 cells. The numbers of apoptotic cells were fewer in resistant breast cancer cells than in controls. Many protein molecules are involved in the apoptotic cell death signaling transduction, including caspases and PARP. Caspases contain initiator caspases (caspase-2, -8, -10, -9) and effector caspases (caspase-3, -6, -7) [29]. These caspases ultimately converge in the activation of caspase-3 and/or-7 for induction of caspase-dependent cell death [30, 31]. Indeed, activated-caspase proteins can promote apoptosis of cancer cells. The active caspase-3 can lead to cleavage of cytoplasmic and nuclear PARP. The cleaved-PARP is also a biomarker of apoptosis [29]. Therefore, we investigated the relationship between lncRNA APOC1P1-3 and the apoptotic proteins, and found that caspase 3, 8, 9 and PARP increased as the lncRNA APOC1P1-3 decreased. Whereas, activated caspase and cleaved-PARP proteins decreased as the lncRNA APOC1P1-3 increased (Fig. 3e, f). All these results suggest that lncRNA APOC1P1-3 could promote anoikis resistance via caspase-dependent pathway.

The lncRNAs could interplay with proteins, DNA, and RNA to regulate biological processes [32]. There is a cross-regulation between lncRNAs and microRNA [33, 34]. LncRNAs interact with microRNA through different pathways. For example, microRNA can influence the stability of lncRNAs. LncRNAs can act as miRNA decoys or sponges to repress target mRNAs [35]. LncRNAs can also compete with miRNAs by directly binding to the shared target mRNAs. In the current study, we screened 84 microRNAs which may bind to lncRNA APOC1P1-3 using RNA microarrays and predicted microRNAs in multiple databases like PITA, MIRDB4.0 and Miranda [36]. We further isolated the lncRNA APOC1P1-3 from cytoplasm and explored its interaction with specific miRNAs. miRNA-188-3p is most likely binding to lncRNA APOC1P1-3 (Fig. 4a) at 222-248 bp of APOC1P1-3 (Fig. 4b, d).

The miRNA-188-3p is a novel independent prognostic factor in colorectal cancer patients because it promotes cancer cell migration [37]. In addition, it negatively regulates NCAPG2 via activating NF-κb to block NCAPG2-mediated hepatocarcinoma proliferation and metastasis [38]. It is also involved in sevoflurane-induced cognitive dysfunction via MDM2 proto-oncogene [39]. Furthermore, it negatively regulates TMED3 to inhibit the proliferation, migration and invasion of breast tumor [40]. Thus, miRNA-188-3p has different functions in diverse pathological conditions. In this study, miRNA-188-3p promotes the early apoptosis of MDA-MB-231a cells, suggesting an inhibition of anoikis resistance. lncRNA APOC1P1-3 could inhibit apoptosis by suppressing the function of miRNA-188-3p.

Bcl-2 proteins, are anti-apoptotic and promote breast cancer formation and progression [41]. We investigated the correlation among the lncRNA APOC1P1-3, miRNA-188-3p and Bcl-2 and found that down-regulation of miRNA-188-3p increases and overexpression decreases Bcl-2 gene expression. This indicates that miRNA-188-3p regulates Bcl-2 at transcription level. Furthermore, overexpression of lncRNA- APOC1P1-3 increases the expression of Bcl-2, but the overexpression of lncRNA- APOC1P1-3 followed by up-regulation of miRNA-188-3p abolishes the effect on Bcl-2. The mechanism of the miRNA-188-3p regulating Bcl-2 is still unclear. Further investigation is needed.

In summary, lncRNA APOC1P1-3 can promote the anoikis resistance of breast cancer cells and specifically bind to miRNA-188-3p acting as a “sponge” to block the Bcl-2 inhibition. Direct or indirect interaction of miR-188-3p and Bcl-2 need to be investigated.

## Supplementary Information

**Additional file 1: Table S1. **Verification of the non-coding characteristics of lncRNA-APOC1P1-3.** Table S2. **Comparison of the target microRNAs with different databases.** Table S3. **20 sets of random mutations for binding sites, and its thermodynamics.** Table S4. **The primer sequences are described in Table S4.** Figure S1.** Thermodynamic analysis of the predicted binding sites by BiBiServ-RNAhybrid.** Figure S2.** Site-directed mutation in pmirGLO-APOC1P1-3’UTR plasmid.

**Additional file 2: Table S5. **The patient information.

## Data Availability

The datasets generated and used in this study are available from the corresponding author on reasonable request.

## Abbreviations

lncRNA APOC1P1-3: Long non-coding RNAs APOC1P1-3
ECM: Extracellular matrix
ORF: Open Reading Frame
polyHEMA: Poly-2-hydroxycthyl methacrylate
FACS: Fluorescence-activated cell sorting
