# Supplementary material for: lncRNA APOC1P1-3 promoting anoikis-resistance of breast cancer cells
Source: Cancer Cell Int. 2021 Apr 26;21:232. doi: 10.1186/s12935-021-01916-w (PMC8074441; doi:10.1186/s12935-021-01916-w)

LncRNA APOC1P1-3 promoting anoikis-resistance of breast cancer cells

Ping Zhou*

Supplementary

Table S1. Verification of the non-coding characteristics of lncRNA-APOC1P1-3.

Label	Strand	Frame	Start	Stop	Length(bp/aa)
ORF6	-	2	453	286	168/55
ORF8	-	3	380	297	84/27
ORF3	+	3	51	125	75/24
ORF2	+	2	428	493	66/21
ORF4	+	3	240	302	63/20
ORF5	-	1	52	＞2	51/16
ORF7	-	2	264	214	51/16
ORF1	+	1	379	420	42/13

LncRNA-APOC1P1-3 has 8 ORFs, each is less than 200nt. All ORFs labeled "+" after BLAST and these short peptides have no homologous proteins.

Table S2. Comparison of the target microRNAs with different databases.

Names	Total	Elements
PITA<br>Kangcheng	8	hsa-miR-193a-5p<br>hsa-miR-587<br>hsa-miR-566<br>hsa-miR-483-5p<br>hsa-miR-188-3p<br>hsa-miR-369-5p<br>hsa-miR-502-5p<br>hsa-miR-1290
Miranda<br>PITA	2	hsa-miR-204<br>hsa-miR-211
Kangcheng<br>miRDB4<br>PITA	1	hsa-miR-188-3p

There are 8 cross results among PITA database and chip results (Kangcheng). PITA and Miranda have 2 cross results, and miRDB4, PITA and Kangcheng have 1 cross result.

Table S3. 20 sets of random mutations for binding sites, and its thermodynamics.

NO.	Mutant sequences	mfe(kcal/mol)
1	AGTGAAGTTCGTGGCAAGATCTGCGAT	-18.6
2	AGTGAGCTTCATACCAAGGTGTGGAAT	-18.9
3	AGTGAATTTGCTGGCAAGCTGTAGGAT	-19.4
**4**	**AGTGAGCTTCGTGCCAAGGTGGGGTAT**	**-25.4**
5	AGTGAGCTTCGTGCCAAGGTGGGGCAT	-25.3
6	AGTGAGCTTCTTGGCAAGGTGTAGGAT	-20.7
7	AGTGGACTTTCTACCAAGCTGTGAGAT	-17.5
8	AGTGGACTTCCGGCCAAGGTGGGCGAT	-22.3
9	AGTGAATTTCATGGCAAGGTGTAGGAT	-18.3
10	AGTGAGCTTACTGTCAAGACGTGCGAT	-17.3
11	AGTGGACTTACTACCAAGGTGCGGGAT	-17.6

Table S4. The primer sequences are described in table S4.

Gene name	Forward primer(5'-3')	Reverse primer(5'-3')
APOC1P1-3 Primer1	GGTCCTGGTGGTGGTTCTGTC	CTCCTTCACTTTCCGAAATGTCTC
APOC1P1-3 Primer2	AGGATTCAGGTTGGTGCCC	TCTGTGTGATGCGGTTGATGA
GAPDH	GGGAAACTGTGGCGTGAT	GAGTGGGTGTCGCTGTTGA
β-actin	GGTGGCTTTTAGGATGGCAAG	ACTGGAACGGTGAAGGTGACAG

Figure S1. Thermodynamic analysis of the predicted binding sites by BiBiServ-RNAhybrid.

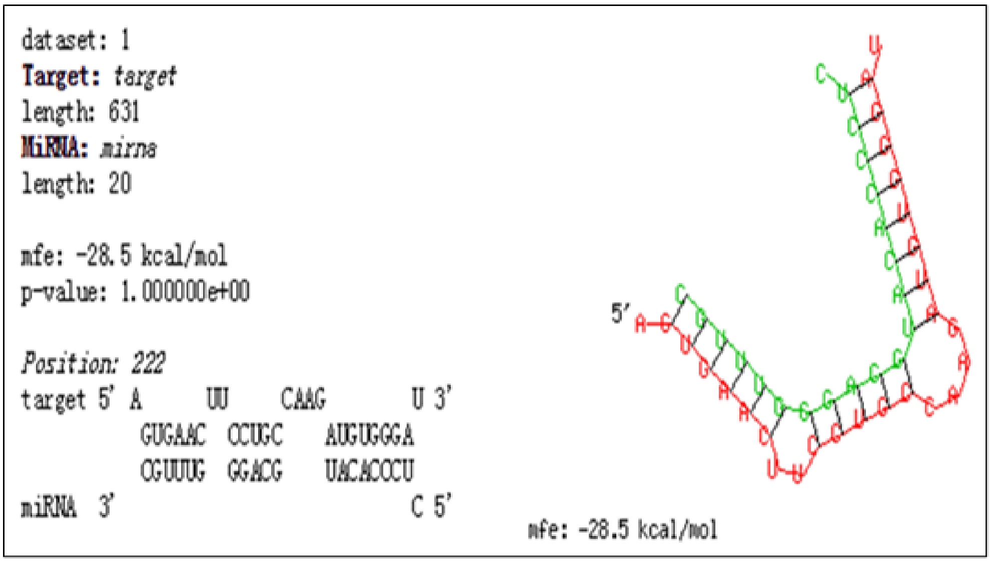

Figure S2. Site-directed mutation in pmirGLO-APOC1P1-3'UTR plasmid.

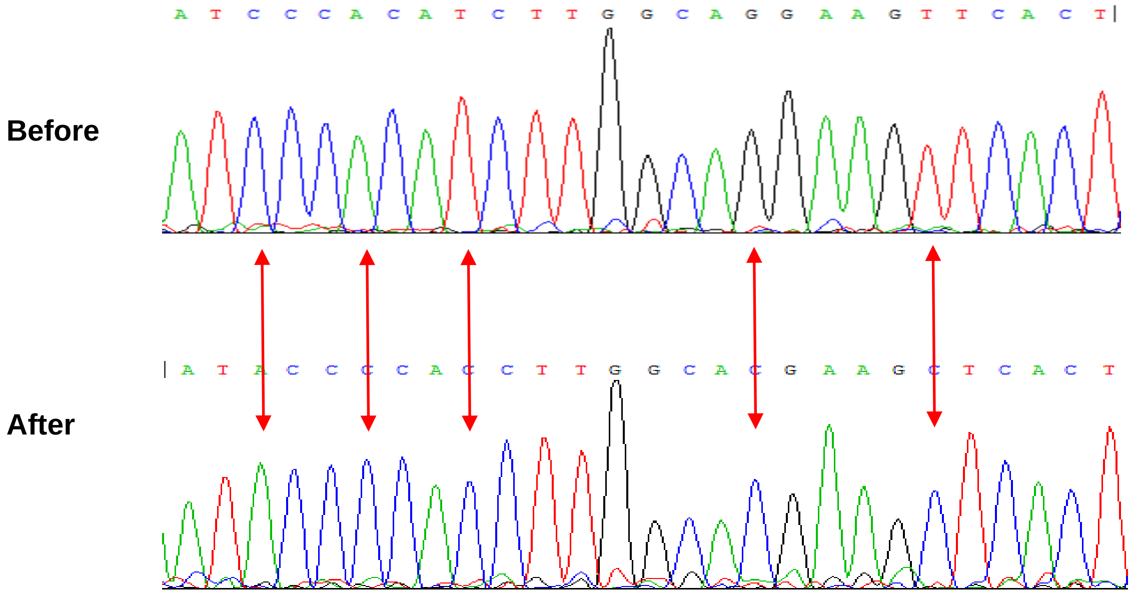

Supplement: Supplementary file 1 — Additional file 1: Table S1. Verification of the non-coding characteristics of lncRNA-APOC1P1-3. Table S2. Comparison of the target microRNAs with different databases. Table S3. 20 sets of random mutations for binding sites, and its thermodynamics. Table S4. The primer sequences are described in Table S4. Figure S1. Thermodynamic analysis of the predicted binding sites by BiBiServ-RNAhybrid. Figure S2. Site-directed mutation in pmirGLO-APOC1P1-3’UTR plasmid. [file 12935_2021_1916_MOESM1_ESM.pdf]
